# Impact of Blood Pressure Self-Management on Vascular Remodeling After Hypertensive Pregnancy

**DOI:** 10.1161/HYPERTENSIONAHA.125.24854

**Published:** 2025-09-04

**Authors:** Jamie Kitt, Luca Biasiolli, Samuel Krasner, Paul A. Bateman, Hannah R. Cutler, Logan C. Barr, Annabelle Frost, Katherine L. Tucker, Katie Suriano, Yvonne Kenworthy, Winok Lapidaire, Miriam Lacharie, Rebecca Mills, Cristian Roman, Lucy Mackillop, Christina Y. L. Aye, Alexandra E. Cairns, Basky Thilaganathan, Lucy C. Chappell, Adam J. Lewandowski, Richard J. McManus, Paul Leeson

**Affiliations:** Oxford Cardiovascular Clinical Research Facility, Division of Cardiovascular Medicine, Radcliffe Department of Medicine (J.K., S.K., H.R.C., A.F., K.S., Y.K., W.L., P.L.), University of Oxford, United Kingdom.; Nuffield Department of Primary Care Health Sciences (J.K., P.A.B., K.L.T., R.J.M.), University of Oxford, United Kingdom.; Oxford Centre for Clinical Magnetic Resonance Research, Division of Cardiovascular Medicine, Radcliffe Department of Medicine (L.B., M.L., R.M.), University of Oxford, United Kingdom.; Institute of Biomedical Engineering, Department of Engineering Science (C.R.), University of Oxford, United Kingdom.; Nuffield Department of Women’s and Reproductive Health (A.F., L.M., C.Y.L.A., A.E.C.), University of Oxford, United Kingdom.; Nuffield Department of Population Health (A.J.L.), University of Oxford, United Kingdom.; Queen’s University School of Medicine, Kingston, Ontario, Canada (L.C.B.).; Fetal Medicine Unit, Oxford University Hospitals National Health Service Foundation Trust, United Kingdom (C.Y.L.A., A.E.C.).; Fetal Medicine Unit, St George's University Hospitals National Health Service Foundation Trust, University of London, London, United Kingdom (B.T.).; Department of Women and Children’s Health, King’s College London, St Thomas’ Hospital, United Kingdom (L.C.C.).

**Keywords:** hypertension, postpartum period, pre-eclampsia, pregnancy, vascular remodeling

## Abstract

**BACKGROUND:**

Hypertensive pregnancy disorders are associated with long-term adverse cardiac and vascular remodeling post index pregnancy. The POP-HT trial (Physician Optimised Postpartum Hypertension Treatment) demonstrated that improved puerperal blood pressure control leads to reduced blood pressure and beneficial cardiac remodeling during the first year postpartum. This study describes the impact on postpartum vascular remodeling.

**METHODS:**

A prospective, randomized, open-label, blinded end point trial in a single UK hospital where 220 women were assigned 1:1 to intervention (self-management via physician-guided antihypertensive titration) or control (usual postnatal care via primary care doctor±midwife). Eligible participants were ≥18 years, with preeclampsia or gestational hypertension and requiring antihypertensives on discharge. Prespecified secondary vascular outcomes included aortic blood pressure and pulse wave velocity measured by Vicorder at baseline and 9 months postpartum, and additional cardiovascular magnetic resonance measures of aortic distensibility were performed.

**RESULTS:**

There were no baseline differences in aortic blood pressure or pulse wave velocity but by 9 months postpartum, aortic diastolic blood pressure was −5.2 mm Hg lower ([95% CI, −8.0 to −2.2]; *P*<0.001), and pulse wave velocity was −0.71 m/s lower ([95% CI, −1.42 to −0.06]; *P*=0.048) in the intervention arm compared with the control arm, which corresponded with greater aortic distensibility by 0.78×10^−^^3^ mm Hg^−^^1^ ([95% CI, 0.01 to 1.55]; *P*=0.046).

**CONCLUSIONS:**

Postpartum blood pressure self-monitoring combined with physician-guided medication titration is associated with reduced central arterial stiffness during the first year after a hypertensive pregnancy, in addition to the previously demonstrated effects on blood pressure and cardiac remodeling.

**REGISTRATION:**

URL: https://www.clinicaltrials.gov; Unique identifier: NCT04273854.

**NOVELTY AND RELEVANCE**

**What Is New?**

The POP-HT (Physician Optimised Postpartum Hypertension Treatment) vascular substudy used multiple measures of aortic stiffness, including measures of aortic blood pressure and pulse wave velocity, as well as aortic magnetic resonance assessment of aortic distensibility, to demonstrate that blood pressure self-management in the postpartum period is associated with improved vascular measures at 9 months postpartum.

**What Is Relevant?**

In addition to previously reported improvements in blood pressure and cardiac remodeling, this study now demonstrates that remote telemonitored self-management of blood pressure postpartum has additional beneficial effects on central arterial stiffness after hypertensive pregnancies.

**What Are The Clinical Implications?**

Given the association of aortic stiffness with an increase in long-term cardiovascular risk and adverse cardiovascular outcomes, including stroke and heart attack, this data provides further evidence that interventions in the postpartum period may be important to reduce the long-term known cardiovascular risks in the large cohort of women affected by hypertensive pregnancy.

Numerous studies have highlighted microvasculature,^1^ conduit,^2^ and large vessel remodeling^1,3,4^ in women who have had a hypertensive pregnancy in the years after pregnancy. A meta-analysis demonstrated that arterial stiffness,^5^ measured by pulse wave velocity (PWV), is elevated 2 years after a hypertensive pregnancy, and we previously reported aortic compliance is lower up to 10 years later.^1^ Adverse vascular phenotypes such as these predict worse longer-term outcomes, such as stroke^6,7^ and a greater incidence of earlier onset heart failure,^8^ which are known to be more common in women who have had hypertensive pregnancies compared with normotensive pregnancy.^6^ Whether these vascular differences reflect changes that were already present before pregnancy or develop, or worsen, during pregnancy remains unclear. However, during both hypertensive and normotensive pregnancies, hemodynamic and hormonal changes lead to extensive vascular remodeling. These changes then need to reverse to prepregnancy patterns during the postpartum period.^8^

Blood pressure levels immediately postpartum are often rapid and unpredictable and typically remain at higher levels for several weeks after a hypertensive pregnancy compared with after a normotensive pregnancy.^9,10^ We hypothesized that poor blood pressure control after a hypertensive pregnancy might limit normal postpartum vascular reverse remodeling and that this may contribute to persistent abnormalities in vascular responses.^11–14^ In the POP-HT (Physician Optimised Postpartum Hypertension Treatment) randomized clinical trial, we demonstrated that physician-guided antihypertensive self-management after hypertensive pregnancy results in lower blood pressure for at least 9 months postpartum.^15^ Participants also underwent multimodality imaging to investigate prespecified secondary imaging outcomes to test the mechanistic hypothesis that blood pressure improvements associate with beneficial cardiac and vascular remodeling. We have previously reported beneficial changes in cardiac parameters,^16^ and now we present the vascular outcome measures.

## Methods

### Data Availability

The data that support the findings of this study are available from the chief investigator (P.L.), on reasonable request, subject to the approval of the sponsor (University of Oxford) and the trial steering committee.

### Study Design and Participants

POP-HT was a single-center, 2-group parallel, PROBE study (Prospective Randomized, Open, Blinded End-Point). Detailed descriptions of the methodology, including recruitment, patient characteristics, and statistical analysis, and prespecified outcome measures, including the secondary imaging have previously been published.^15,17^ In brief, all participants were recruited from the Women’s Centre at Oxford University Hospitals National Health Service Foundation Trust in the United Kingdom. Participants were aged ≥18 years, with a clinician-confirmed diagnosis of either gestational hypertension or preeclampsia according to UK National Institute for Health and Care Excellence guidance,^12^ and still required antihypertensive medication at the time of hospital discharge. Participants with chronic/essential hypertension, defined as a blood pressure >140/90 mm Hg at their 12-week booking assessment, or those already on antihypertensive treatment before pregnancy, were excluded.

Participant information on race and ethnicity was self-reported using the UK Office of National Statistics prespecified categories (people and places: ethnicity and race–content style guide–service manual–Office for National Statistics). Individuals with: hypertension before pregnancy, medical conditions that made self-monitoring impractical or unsafe, those unable to follow the English POP-HT study application (app)–based instructions, and those unable to provide written consent were excluded. The trial was supervised by a trial steering and data safety monitoring committee. Ethical and research governance approvals were gained from the London-Surrey Research Ethics Committee (Reference 19/L0/1901, Integrated Research Approval System Project ID: 273353) and the local hospital trust.

### Randomization and Blinding

After a baseline visit, eligible participants were randomized 1:1 to either telemonitored home blood pressure monitoring with physician-assisted self-management, or standard National Health Service–led care from their obstetric team, primary care practitioner, and midwives. Randomization was conducted with secure web-based software (Castor Electronic Data Capture) with minimization for gestational age, whether the patient had a diagnosis of preeclampsia or gestational hypertension, and prescription of an angiotensin-converting enzyme (ACE) inhibitor at the time of randomization. Due to the nature of the intervention, participants could not be blinded to group assignment, but given this was a PROBE study, investigators were blinded to group assignment during data analysis.

### Intervention and Study Visits

**Figure 1.**
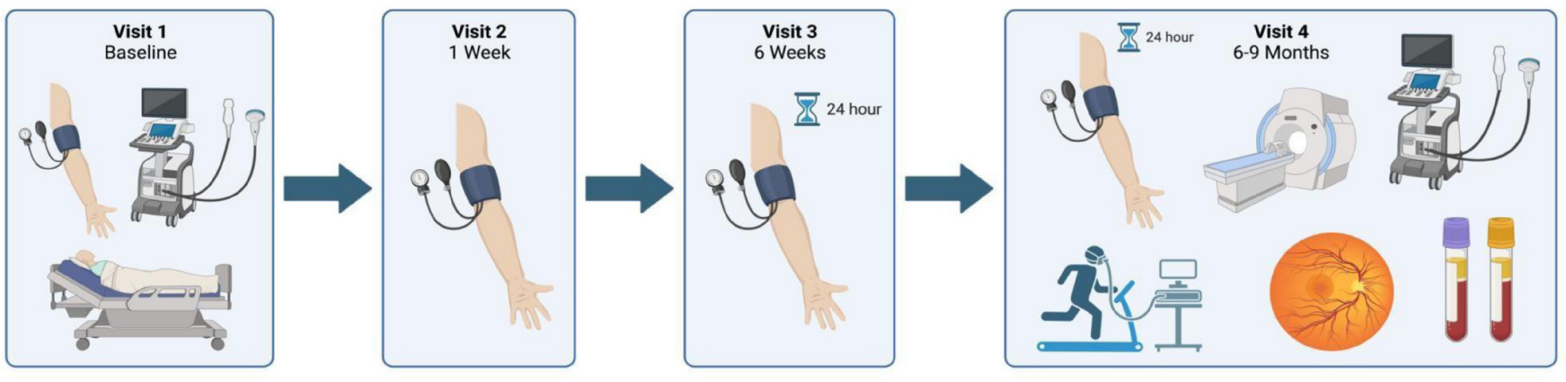
**Flow diagram of study visits from baseline visit (visit 1 [V1]) to final study visit (visit 4 [V4]).** V1/baseline visit took place on postnatal ward at D1−D5 postpartum and included transthoracic echocardiogram, Vicorder assessment of aortic blood pressure, pulse wave velocity and augmentation index, peripheral blood pressure measurement, and anthropometric measures, as well as documentation of relevant obstetric and medical history, medications, and blood and urine results. Visit 2 took place at week 1±5 days postpartum and included an update of the medical and obstetric history, medication doses and any side effects, any new investigations/admissions, and measurement of peripheral blood pressure using a validated, calibrated monitor. Visit 3 took place at week 6±5 days postpartum and included an update of the medical and obstetric history, medication doses and any side-effects, any new investigations/admissions, and measurement of peripheral blood pressure using a validated, calibrated monitor, and a 24-hour ambulatory blood pressure monitoring was fitted and posted back to the study site for analysis. V4/Final study visit took place at ≈9 months postpartum (249.8 days [14.6] in intervention arm vs 247.9 days [17.1] in the control arm) and included all measures performed at V1 plus magnetic resonance imaging of brain, heart, kidneys and aorta, retinal imaging, repeated blood sampling for lipid and sugar metabolism and assessment of renal function and a cardiopulmonary exercise test and bicycle exercise echocardiogram. Created in BioRender. Leeson, P. (2025) https://BioRender.com/4d0xcpu.

Participants assigned to the usual care arm were discharged from the hospital for ongoing management according to local standard care. National UK guidance recommends standard care as a minimum of a blood pressure review with a primary care practitioner or community midwife at day 1 to 14 postpartum, a 2-week review with their family physician, and a 6 to 8 week review with their family physician or specialist.^12^ Titration of antihypertensive treatment was at the discretion of their supervising healthcare professionals (primary care physician and midwife). Participants in the intervention group had initial discharge medications decided by their clinical care team,^12^ and then dose titration after hospital discharge was guided remotely by the research team physicians, including cardiologists and obstetricians, in response to daily self-monitored blood pressure measurements (increased to twice daily if out of target range; see published protocol paper for further details).^15,17^ Choice of medication and titration regimes was standardized based on recommendations from the 2019 UK National Institute for Health and Care Excellence guidance for management of pregnancy hypertension.^12^ There were 4 study visits after prescreening enrollment, occurring at days 1 to 6 postpartum (visit 1 [V1]; baseline), 1 week (visit 2), 6 weeks (visit 3), and 6 to 9 months (visit 4 [V4]). This is summarized in Figure 1.

### Outcome Measures

#### Aortic Blood Pressure and PWV

Participants in both groups had research measurements of clinic blood pressure at each study visit, and the primary outcome of the trial was 24-hour mean diastolic blood pressure, measured by ambulatory blood pressure monitoring (model 90217; Spacelabs Healthcare, Snoqualmie) at the time of the final study visit (V4).^18^ All participants were also invited to have a noninvasive Vicorder assessment of aortic blood pressure, and PWV (m/s; Skidmore Medical, United Kingdom) at baseline (V1) while on the postnatal ward.^17^ Brachial and femoral cuffs were fitted (a known measured distance apart) and connected to an oscillometric device (Vicorder). The cuffs were then partially inflated and used to detect pulse arrival and thus derive PWV based on the distance between cuffs. Brachial artery pressure waveform was also used to derive estimates of central blood pressure and augmentation index. Measurements were performed in a standardized way with the participant reclined to a supine position in electronic hospital beds. Participants were also offered the same Vicorder protocol when attending their final study visit (V4) in the Cardiovascular Clinical Research Facility at the John Radcliffe Hospital, Oxford, United Kingdom.

#### Aortic Distensibility

A separate, prespecified cardiovascular magnetic resonance (CMR) study was also offered to participants at V4. This, in addition to previously reported cardiac outcomes,^16^ included images of their ascending aorta at the main pulmonary artery level. Scans were performed in the Oxford Centre for Clinical Magnetic Resonance Research using an 18-channel body coil and spine array using CMR (3T PRISMA MR scanner; Siemens Healthineers, Erlangen, Germany) and previously described techniques.^19^ In brief, a retrospective ECG-gated balanced steady-state free precession sequence at the end of exhalation was used to acquire cross‐sectional cine images of the thoracic aorta at the level of the pulmonary artery bifurcation, in the ascending aorta. The maximal and minimal aortic areas were measured using edge detection^19^ within an image analysis tool^20^ (developed in MATLAB, Mathworks Inc, MA) that can also provide an automated image quality assessment of the entire series of cine frames in the region of interest. Compliance was calculated by dividing the change in the aortic area by minimum aortic area. Meanwhile, distensibility was calculated by dividing compliance by 24-hour overall average pulse pressure derived from 24-hour ambulatory blood pressure monitoring data fitted after the CMR study. Study visits were undertaken during the COVID-19 pandemic between 2020 and 2021, and during this time, Vicorder measures were not always available at the time of the CMR study, which was sometimes arranged separately from the clinical follow-up visit. To ensure consistent measures of resting blood pressure for distensibility calculations, we therefore used 24-hour overall average ambulatory measures. Vicorder-derived compliance measures in the subset of participants are included in the Supplemental Material for completeness.

### Statistical Analysis

Analysis was based on the principles of intention-to-treat, including all participants with at least 1 postrandomization outcome. Mean differences between groups with 95% CI and *P* values were estimated from adjusted linear regression models at a single time point (V4) with adjustment for the prespecified minimization factors stated in the statistical analysis plan. The level of statistical significance was tested at a 5% 2-tailed significance level (*P*<0.05). Differences in imaging-based secondary outcomes between groups were evaluated using an adjusted linear regression model, including baseline postnatal measures from V1 for Vicorder.

PWV at the final study visit (V4) was adjusted for baseline measures and aortic diastolic blood pressure at the time of the final study visit. For aortic cine sequences of the ascending aorta, no baseline (V1) measures were available. Therefore, models were adjusted for blood pressure at the time of the CMR study, as well as body surface area and age at the time of the final study visit (V4). Continuous outcome variables were normally distributed according to the Central Limit Theorem. Assumptions of normality and constant variance in linear regression models were confirmed post hoc using residual and other diagnostic plots and not violated. Differences in aortic distensibility were determined by 2-sample *t* test with equal variances after confirming the normality of distribution. Sensitivity analyses were performed using antenatal booking blood pressure in place of baseline postpartum blood pressure, and further post hoc analyses were done by removing those remaining on antihypertensive treatment at the final study visit (V4). No adjustment was made for multiple testing. Analysis was done using STATA Version 14.2, (StataCorp).

## Results

### Study Population

#### Demographics

Between February 21, 2020 and March 21, 2021, 220 participants were enrolled in the study, with 112 assigned to the intervention arm and 108 to the usual care (control) arm. Of 220 participants, baseline Vicorder measures were available for 219 of 220, and 194 of the 202 still enrolled at the final follow-up visit (102 intervention; 92 standard care). The demographic characteristics of the whole study group have been reported previously.^15^ Those who had assessment of vascular measures, as a whole, and according to randomization, are presented in Table 1.

**Table 1.**
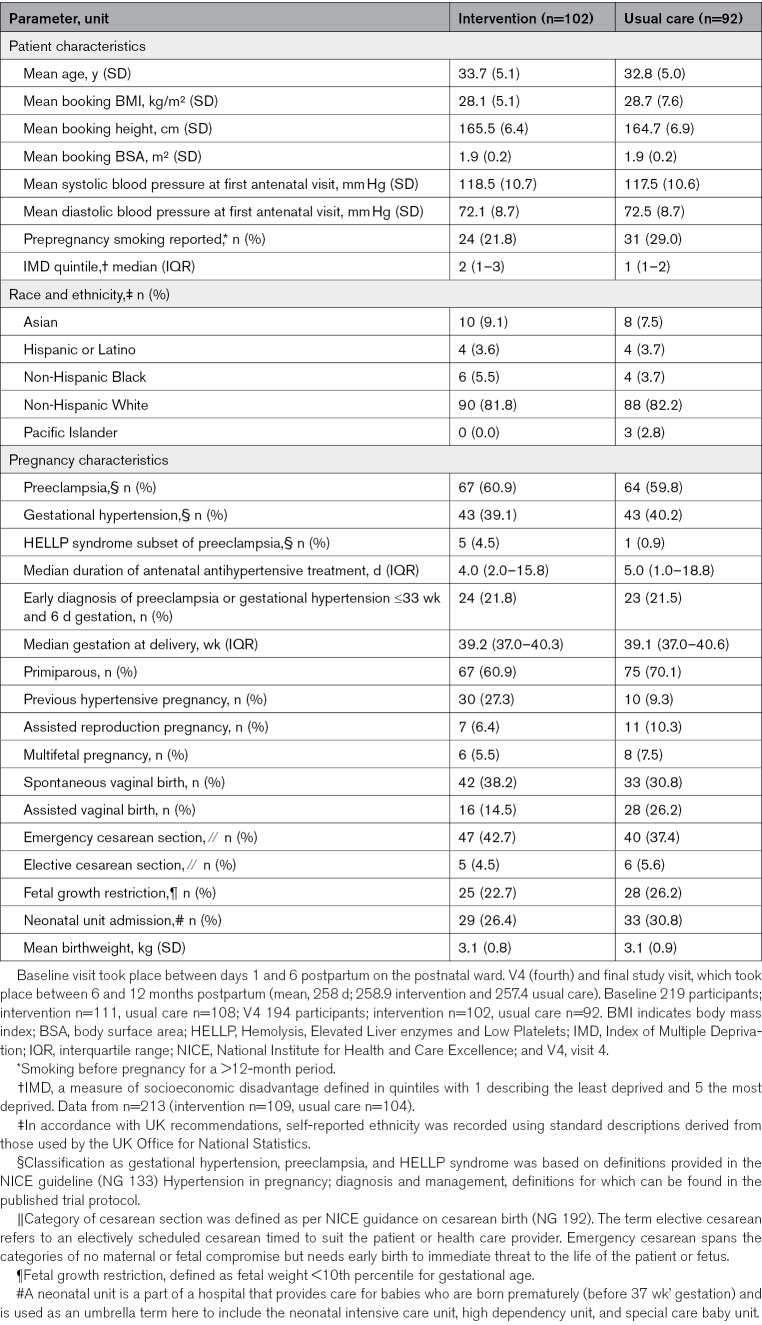
Characteristics of Participants Who Had Vicorder Assessment at Baseline and V4

#### Pregnancy Characteristics

Approximately 40% of those undergoing vascular measures had gestational hypertension, and ≈60% had preeclampsia. As such, there was a higher number with the preeclampsia subtype of hypertensive pregnancy. This is consistent with the inclusion criteria of the trial requiring ongoing antihypertensive treatment at the time of hospital discharge, and as this phenotype is often more severe, women often required medication postnatally to control their blood pressure. The 2 groups were similar in obstetric and pregnancy characteristics at baseline (Table S1), except a higher proportion of participants had a prior hypertensive pregnancy in the intervention arm. Diet and lifestyle characteristics of participants at the time of their vascular assessment were also statistically similar by χ^2^ analysis (Table S2).

#### Antihypertensive Use

Antihypertensive prescription by classes was similar in each group (enalapril 57%, nifedipine 27%, labetalol 30% for intervention versus enalapril 43%, nifedipine 30% and labetalol 27% for usual care). At 6 weeks postpartum (visit 3), ≈30% of participants in each group were still on medication, which reduced to ≈12% by the final visit (V4). Participants in the intervention group were medicated for a median of 39 days (interquartile range, 13.9–41.5 days). Amount of antihypertensives prescribed, defined by the median World Health Organization defined daily dose,^21^ was similar between groups at the baseline visit (V1) and 9 months (V4). However, at visit 2 (week 1), more antihypertensives were prescribed (World Health Organization defined daily dose 1.5 versus 0.7; *P*=0.01) in the intervention group. The characteristics of medication use in those undergoing imaging measures were similar to those observed in the whole trial cohort (Table S3).

### Aortic Blood Pressure and PWV

**Figure 2.**
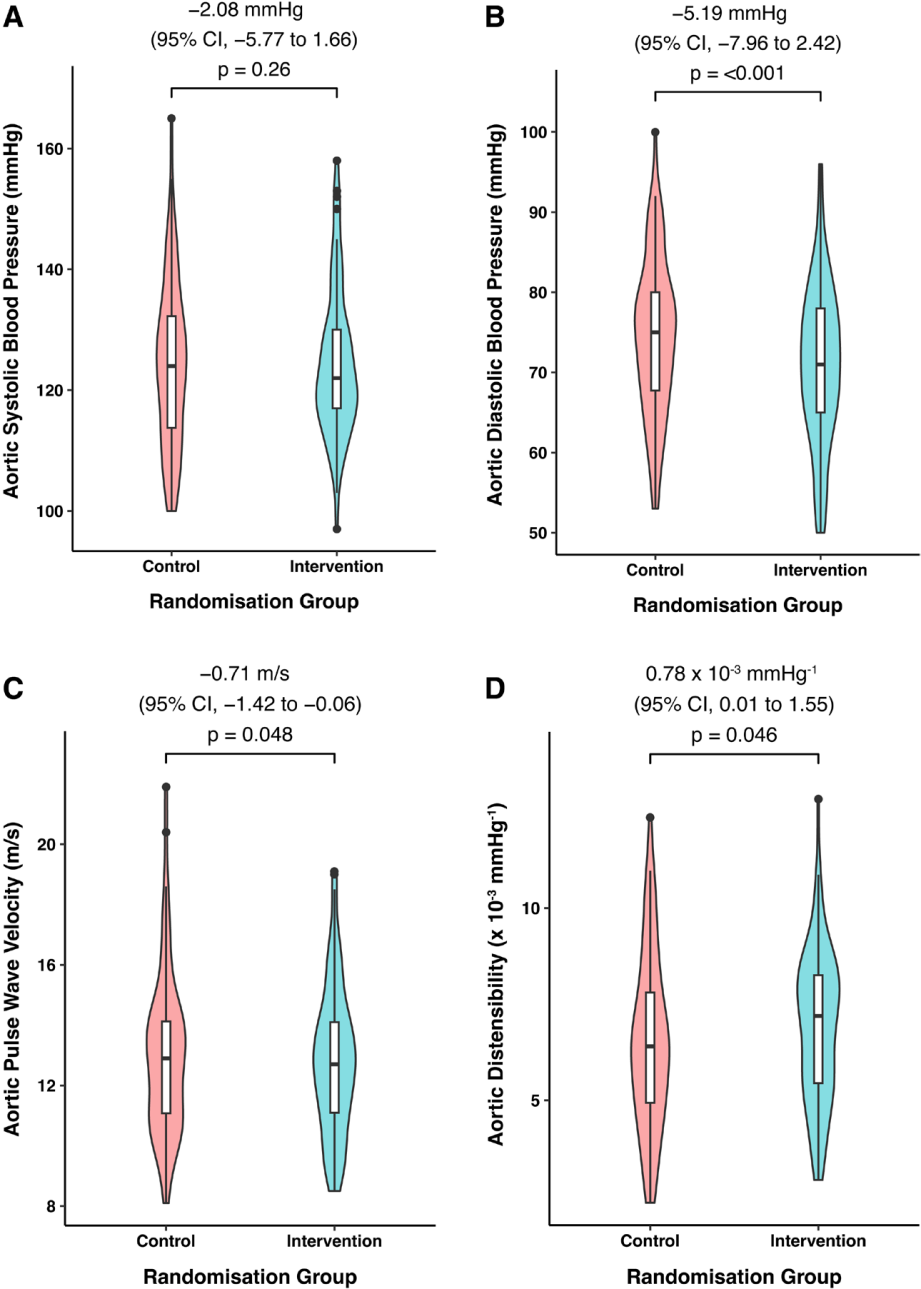
**Aortic systolic and diastolic blood pressure (BP), aortic pulse wave velocity, and aortic distensibility at ≈9 months postpartum by randomization group. A**, Aortic systolic BP at ≈9 months postpartum adjusted for baseline differences. **B**, Aortic diastolic BP at ≈9 months postpartum adjusted for baseline differences. **C**, Aortic pulse wave velocity (m/s) adjusted for aortic diastolic BP at time of the visit 4 (V4) assessment. **D**, Aortic distensibility at ≈9 months postpartum. Violin plots with overlaid box plots. Tukey box plots represent median and interquartile range (IQR), whiskers represent largest value within 1.5× IQR above 75th percentile and smallest value within 1.5× IQR below 25th percentile, and data points beyond the whiskers represent values >1.5× and <3× the IQR. Adjusted mean difference, 95% CI, and *P* values are provided above each plot, representing the significance between control and intervention groups at 9 months. Linear regression models on the Vicorder data were performed on Vicorder measures from V4 with measures from the baseline visit/visit 1 (V1) and baseline mean diastolic BP included in the model as per the original study Statistical Analysis Plan. For pulse wave velocity, data were adjusted for aortic diastolic BP at the time of V4 and baseline Vicorder measures. Aortic distensibility is adjusted for body surface area (calculated by Mosteller equation) and age at the time of final study visit. Aortic distensibility (10^−3^ mm Hg^−1^) was calculated as follows: (A_max_–A_min_)/A_min_×(P_max_–P_min_), where A_max_ and A_min_ represent the maximal and minimal cross-sectional area of the aorta on cine cardiovascular magnetic resonance images, and P_max_ and P_min_ represent the V4 24-hour averaged systolic and diastolic BP (in millimeters of mercury), respectively.

There were no baseline differences in Vicorder-derived measures of aortic blood pressure or arterial function performed on the postnatal ward at day 1 to 6 postpartum. By a median of 258 days postpartum (259±7 days for the intervention arm and 257±8 days for the usual care arm), aortic diastolic blood pressure was a mean 5.2 mm Hg lower in the intervention arm ([95% CI, −8.0 to 2.2]; *P*<0.001) compared with the control group. At the same time point, PWV adjusted for aortic diastolic blood pressure and baseline differences was 0.71 m/s lower ([95% CI, −1.4 to −0.006]; *P*=0.048) in the intervention group compared with control There was no significant difference in aortic systolic blood pressure or augmentation index as shown in Table 2 and Figure 2 (mean difference, −2.08 mm Hg [95% CI, −5.77 to 1.66]; *P*=0.26).

**Table 2.**
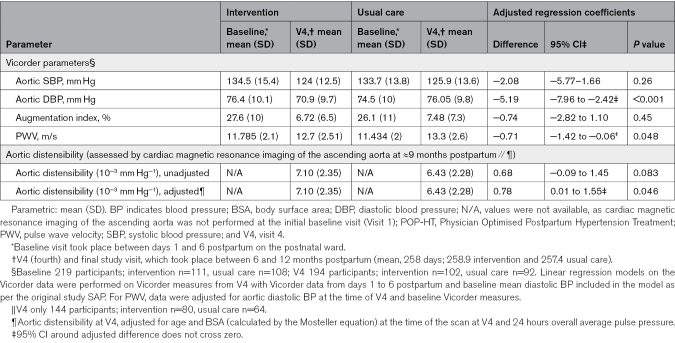
Aortic (Vascular) Remodeling Data From the POP-HT Randomized Trial

### Magnetic Resonance Imaging of the Ascending Aorta

CMR scans were obtained in 174 participants at V4, of which 93 were in the intervention group and 81 in the usual care group. Demographics of all participants randomized in the subgroup who had CMR-derived aortic distensibility measures are presented in Table S1 and are similar to those presented in Table 1. High-quality CMR-derived aortic distensibility scans were acquired in 145 participants (80 intervention, 65 control). The remainder were rejected by automated assessment of the cine image quality at the level of the ascending aorta, which was confirmed by visual review (L. Biasiolli). Reasons for exclusion included severe imaging artifacts (due to patient movement/breathing, as well as artifacts from breast implants), off-plane sequences, or an inadequate number of cine frames being acquired during the breath-hold. The results demonstrate a greater aortic distensibility within the intervention group compared with the control group, when adjusted for age and body surface area at the time of the CMR scan,^20^ of 0.78×10^−^^3^ mm Hg^−^^1^ ([95% CI, 0.01×10^−^^3^ to 1.55×10^−^^3^]; *P*=0.046; Figure 2).

### Additional Analyses

Unadjusted PWV and aortic distensibility differences are reported in Table S4. Additional post hoc sensitivity analyses were performed to investigate the relevance of antihypertensive treatment at the time of V4. Results after exclusion of the 24 participants still on blood pressure–lowering medication at the time of their CMR (n=16 in the intervention arm and n=8 in the control arm) are shown in Table S5. This demonstrated no significant impact on vascular function, consistent with the peripheral vascular/blood pressure results published previously.^15^ Aortic distensibility calculated using pulse pressure obtained from the Vicorder measures taken during their final study visit (V4) is also included in Table S6. This also demonstrated an improvement in aortic distensibility in the intervention arm, consistent with the findings shown in Figure 2 (which uses pulse pressure from 24-hour ambulatory blood pressure monitoring data).

## Discussion

This study demonstrates postpartum blood pressure self-monitoring combined with physician-guided medication titration is associated with reduced central arterial stiffness in the first year after a hypertensive pregnancy, when compared with usual care. These findings extend our previous observation of a beneficial impact of postpartum blood pressure self-monitoring and antihypertensive titration on blood pressure and cardiac remodeling.^15,16^

Hypertensive disorders of pregnancy and a range of cardiovascular diseases share patterns of vascular dysfunction involving endothelial, inflammatory, and angiogenic pathways.^3^ These pathways are known to impact a range of different vascular measures, including central aortic function, and we have previously shown aortic distensibility is reduced for up to 10 years after a hypertensive pregnancy.^4^ Aortic stiffness is greater in those with cardiovascular risk factors and has a particularly close and complex relationship with blood pressure.^22^ Blood pressure control is both worsened by reduced aortic compliance and contributes to the vascular damage that stiffens the aorta.^22–24^ Later evidence of aortic stiffening in those with a hypertensive pregnancy may therefore be secondary to sustained hypertension. However, aortic measures are also an independent predictor of cardiovascular outcome^25–28^ and changes in aortic stiffness early after pregnancy, or interventions that improve aortic stiffness at this time, such as we have demonstrated here, may have important implications for both future development of hypertension and risk of later vascular diseases.

A strength of this study is that current gold-standard measures (Table S7) of aortic stiffness were used,^29^ specifically PWV, which reflects the time taken for a pressure or flow wave to travel along the aorta.^30–32^ Validation studies for the Vicorder device were undertaken in populations approximately age-matched to our trial participants, suggesting that our measures are likely to reflect changes in arterial stiffness seen in this age group.^33,34^ We also used different models to adjust for blood pressure indices that are known to be coassociated with changes in PWV to ensure reported differences reflect changes in aortic function rather than blood pressure variation. The findings are further supported by alternative methods for evaluation of aortic stiffness, specifically, CMR-derived aortic distensibility,^22^ with similar group differences in arterial stiffness observed.

Prior work in young adults has shown that ambulatory blood pressure during waking hours is related to both left ventricular and arterial function. The strongest relations are between ambulatory diastolic blood pressure and left ventricular function, while ambulatory systolic blood pressure is most closely associated with arterial stiffness.^35^ In POP-HT, both daytime and nocturnal systolic and diastolic blood pressure improved after the intervention when assessed by 24-hour ambulatory blood pressure monitoring.^15^ Previously, we reported changes in stroke volume by echocardiography and CMR,^16^ and diastolic function using echocardiography, consistent with the lower diastolic blood pressure seen in the intervention group in POP-HT. This vascular substudy now confirms that there are additional benefits on vascular function, which may contribute to the lower systolic blood pressure previously described.^15^ Strikingly, reductions in blood pressure after postpartum blood pressure self-management were evident even in women who had ceased taking medication, and, in previous long-term follow-up of a small pilot study, such benefits remained evident up to 4 years after delivery.^36^ These persistent blood pressure effects, independent of medication or lifestyle, would be explained by the more beneficial reverse remodeling of both heart and blood vessels achieved by the self-management intervention.

### Limitations

This study has limitations. First, due to the self-monitoring during the intervention, the study was unblinded. However, standardized protocols and practices were followed to minimize potential bias, and the CMR aortic distensibility analysis was done blinded to allocation. Second, the study was impacted by COVID-19, and amendments to the protocol were required to allow for remote study visits. Additionally, some participants were not able or willing to attend the CMR follow-up. Measures of central aortic blood pressure were also not consistently available at the time of the CMR study. However, loss to imaging follow-up remained small (<10%), and there were no significant differences in characteristics between the full cohort,^12^ and those who had CMR scans. Third, the majority of participants were of white British ethnicity, due to the nature of local population demographics.^37^ Additional work is required to understand whether similar patterns of vascular remodeling in response to the intervention are seen in other ethnic and geographic groups. Fourth, those with chronic hypertension were excluded, and so the impact of preexisting hypertensive cardiac and vascular remodeling on both the pregnancy response and postpartum remodeling has not been explored. Finally, the sample size did not allow for study of the impact of different medications on postpartum outcomes. Half of the participants received enalapril in each arm, and there was also similar use of labetalol and calcium channel blockers between arms.^12^ Further clinical and experimental studies will be valuable to understand whether targeted lifestyle or medication approaches, including novel agents such as SGLT-2 (sodium-glucose cotransporter 2) inhibitors, may provide additional beneficial effects.

### Conclusions

This prespecified secondary analysis of the POP-HT randomized trial shows that, in addition to previously reported improvements in blood pressure and cardiac remodeling, remote monitoring and blood pressure self-management in the immediate postpartum period also have benefits on central arterial stiffness for up to 9 months postpartum after hypertensive pregnancy.

### Perspectives

The POP-HT trial is a randomized clinical trial in 220 women that investigates the value of telemonitored, doctor-guided blood pressure self-management postpartum. The aim was to see whether better blood pressure control postpartum had long-term benefits for cardiovascular health. The primary findings of the trial were that the intervention improves blood pressure control both during the period of intervention and that this benefit remains evident for up to 9 months after delivery, even when the woman has stopped taking their antihypertensives.^12^ The blood pressure changes were also associated with beneficial cardiac remodeling during this time period.

We now report the impact of this intervention on prespecified vascular measures of arterial stiffness, measured by PWV and aortic distensibility. The findings described in this paper suggest improved blood pressure control postnatally may help reverse the adverse vascular remodeling known to occur after a hypertensive pregnancy,^4^ and that these benefits persist for at least 9 months postpartum. As blood pressure and arterial stiffness are closely associated, this vascular remodeling may contribute to the sustained benefits in blood pressure seen in the intervention group. In addition, given the long-term data, which demonstrates that aortic stiffness is an important independent predictor of future cardiovascular outcomes,^25,26,38^ this study provides further evidence that the puerperium may be a time-critical window for intervention in women affected by hypertensive pregnancy to improve their long-term cardiovascular health.

## ARTICLE INFORMATION

### Acknowledgments

The authors acknowledge those who contributed to the POP-HT study (Physician Optimised Postpartum Hypertension Treatment). J. Kitt, A.J. Lewandowski, R.J. McManus, K.L. Tucker, A.E. Cairns, K. Suriano, C.Y.L. Aye, A. Frost, W. Lapidaire, C. Roman, L. Mackillop, L.C. Chappell, B. Thilaganathan, C. Douglas, and P. Leeson contributed to the design of the study. J. Kitt, A.J. Lewandowski, R.J. McManus, and P. Leeson secured funding. J. Kitt, A.J. Lewandowski, R.J. McManus, Y. Kenworthy, A. Frost, K. Suriano, L. Mackillop, W. Lapidaire, and P. Leeson refined the overall study protocol and led the project delivery. B. Thilaganathan, C. Douglas, and L.C. Chappell provided guidance and external refinement. J. Kitt, A.J. Lewandowski, R.J. McManus, L. Mackillop, P. Leeson, and C. Roman designed the intervention. J. Kitt and C. Roman oversaw the delivery of the intervention supported clinically by L. Mackillop, A. Frost, and C.Y.L. Aye. A.J. Lewandowski, W. Lapidaire, J. Kitt, R. Mills, M. Lacharie, and P. Leeson contributed to the development of the magnetic resonance imaging (MRI) protocols. J. Kitt, M. Lacharie, R. Mills, L.C. Barr, W. Lapidaire, and A.J. Lewandowski contributed to MRI image acquisition and quality control. L. Biasiolli performed the analysis and review of aortic cine data. P.A. Bateman performed the statistical analysis. S. Krasner and H. Cutler produced the tables and figures for the main article and Supplemental Material. All authors contributed to the manuscript, read, and accepted the final manuscript.

### Sources of Funding

The study was funded by a British Heart Foundation (BHF) Clinical Research Training Fellowship to J. Kitt, University of Oxford (BHF Grant number FS/19/7/34148) with additional support from the National Institute for Health and Care Research (NIHR) Oxford Biomedical Research Centre and Oxford BHF Centre for Research Excellence. P. Leeson, University of Oxford, and C. Roman, University of Oxford, are supported by the Oxford NIHR Biomedical Research Centre. A.L. Lewandowski, University of Oxford, was supported by a BHF Intermediate Research Fellowship. R. McManus, University of Oxford, and K.L. Tucker, University of Oxford, are supported by the NIHR Oxford and Thames Valley Applied Research Collaboration. R.J. McManus and L.C. Chappell, King’s College London, are NIHR Senior Investigators. The funders of this study had no role in study design, data collection, analysis, interpretation, or writing of the report.

### Disclosures

L. Mackillop is a part-time employee of Optum UK. P. Leeson is a founder and shareholder of a healthcare imaging company and a named inventor on patents related to cardiovascular imaging. R.J. McManus has received blood pressure monitors for research from Omron and has worked with Omron and Sensyne on telemonitoring interventions for which licensing and consultancy fees have been paid to the University of Oxford. C. Roman has received consultancy fees from Sensyne Health for work on telemonitoring products. J. Kitt is an executive committee member of the British Society of Cardiac Imaging and Cardiac Computed Tomography.

### Supplemental Material

Appendix providing additional details and illustrations of the vascular phenotyping procedures described in the methods section

Tables S1–S7

Figures S1–S3

Supplemental References

## Supplementary Material


