# Supplementary material for: Impact of Blood Pressure Self-Management on Vascular Remodeling After Hypertensive Pregnancy
Source: Hypertension. 2025 Sep 4;82(11):1938–47. doi: 10.1161/HYPERTENSIONAHA.125.24854 (PMC12529981; doi:10.1161/HYPERTENSIONAHA.125.24854)

**Online Supplement: Impact of Blood Pressure Self-Management on vascular remodeling after hypertensive pregnancy**

Dr Jamie Kitt^1,2^ DPhil, Dr Luca Biasiolli^1,4^ DPhil, Mr Samuel Krasner BA^1^, Dr Paul A Bateman PhD^2^, Ms Hannah Cutler^1^ MSc, Dr Logan Barr^3^ PhD, Dr Annabelle Frost^1,6^, MBBS, Dr Katherine Tucker^2^ PhD, Dr Katie Suriano^1^ PhD, Mrs Yvonne Kenworthy^1^ BSc, Dr Winok Lapidaire^1^ PhD, Ms Miriam Lacharie^4^ MSc, Mrs Rebecca Mills^4^ MSc, Dr Cristian Roman^5^ PhD, Dr Lucy Mackillop^6^ MA, Dr Christina Aye^6,7^ DPhil, Dr Alexandra Cairns^6,7^ D Phil Professor Basky Thilaganathan^8^ PhD, Professor Lucy C Chappell^9^ PhD, Dr Adam J Lewandowski^10^ DPhil, Professor Richard J McManus^2^ PhD and Professor Paul Leeson^1^ PhD

^1^Division of Cardiovascular Medicine, Radcliffe Department of Medicine, University of Oxford, Oxford, UK.

^2^Nuffield Department of Primary Care Health Sciences, University of Oxford, Oxford, UK

^3^Queen’s University School of Medicine, Kingston, Ontario, Canada.

^4^Oxford Centre for Clinical Magnetic Resonance Research, University of Oxford, Oxford, UK.

^5^Institute of Biomedical Engineering, Department of Engineering Science, University of Oxford, Oxford, UK.

^6^Nuffield Department of Women's and Reproductive Health, University of Oxford, Oxford, UK

^7^Fetal Medicine Unit, Oxford University Hospitals NHS Foundation Trust, Oxford, UK.

^8^Fetal Medicine Unit, St George’s University Hospitals NHS Foundation Trust, UK and Molecular and Clinical Sciences Research Institute, St George’s University of London, UK.

^9^King’s College London and Honorary Consultant Obstetrician at Guy's and St Thomas' NHS Foundation Trust, London, UK.

^10^Nuffield Department of Population Health, University of Oxford, Oxford, UK.

Correspondence to:

Professor Paul Leeson, Oxford Cardiovascular Clinical Research Facility, Division of Cardiovascular Medicine, Radcliffe Department of Medicine, University of Oxford, John Radcliffe Hospital, Oxford. OX3 9DU, UK.

Tel: +44 1865 572833. Fax +44 1865 221111. E-mail: [paul.leeson@cardiov.ox.ac.uk](mailto:paul.leeson@cardiov.ox.ac.uk)

**Appendix of the procedures described in the methods section of the main paper**

Pulse wave analysis and velocity measurement

Pulse wave velocity was measured between brachial and femoral arteries. The validated Vicorder® system (SMT Medical, formally Skidmore Medical, Taunton, UK) was used to obtain pressure waveforms of the brachial and femoral pulse.^1-3^ The Brachial artery pressure waveform was used to derive central blood pressure as illustrated in the figure below. This first measurement based on the brachial artery waveform provides both aortic systolic and diastolic blood pressure values (see figure S1 below). For brachial-femoral pulse wave velocity both a brachial and femoral cuff are required. Both cuffs are fitted (a known measured distance apart) and connected to the oscillometric device (Vicorder®) with the patient lying supine. The cuffs are then partially inflated and used to detect pulse arrival and thus derive pulse wave velocity from the difference between the cuffs (see figure S2). This technique was used during the baseline visit on the postnatal ward with women reclined to supine using their electronic hospital beds to allow standardisation of the procedure, and repeated at the final study visit (V4) at ~9 months postpartum in the same standardised manner.

Aortic distensibility

Pulse wave velocity (PWV) is inversely related to aortic distensibility. This section expands upon the details provided in the methodology section of the main paper. Dedicated magnetic resonance imaging of their ascending aorta at the main pulmonary artery level was performed in the Oxford Centre for Clinical Magnetic Resonance Research. This was performed with an 18-channel body coil and a spine array. Aortic Distensibility Aortic structure and function were assessed using 3T PRISMA® MR scanner (Siemens Healthineers®, Erlangen, Germany). In brief, a retrospective ECG gated steady-state free precession sequence at end breath hold was used to acquire cross‐sectional cine images of the thoracic aorta at the level of the pulmonary artery bifurcation, in the ascending aorta (AA. The maximal and minimal aortic areas were measured using edge detection in the automated image analysis software (Matlab, Mathworks, Inc, MA) developed by Biasiolli et al and validated on the UK BioBank dataset.^4^ The software provided an automated image quality assessment of the entire series of cine frames in the region of interest of AA. Compliance was calculated by dividing the change in aortic area by minimum aortic area. Distensibility was calculated by dividing compliance by pulse pressure (PP in mmHg) using both 24hr ABPM derived PP as shown in Table 2 of the main paper, and separate distensibility analysis was performed using the Vicorder derived central pulse pressure (CPP in mmHg) as shown in Table S5 of this supplementary material.

**Online Supplement Reference list:**

1. Laurent, S. and P. Boutouyrie, Recent advances in arterial stiffness and wave reflection in human hypertension. Hypertension, 2007. 49(6): p. 1202-1206.

2. Hickson, S.S., et al., Validity and repeatability of the Vicorder apparatus: a comparison with the SphygmoCor device. Hypertens Res, 2009. 32(12): p. 1079-85.

3. McEniery, C.M., et al., Normal vascular aging: differential effects on wave reflection and aortic pulse wave velocity: the Anglo-Cardiff Collaborative Trial (ACCT). J Am Coll Cardiol, 2005. 46(9): p. 1753-60.

4. Biasiolli L, Hann E, Lukaschuk E, Carapella V, Paiva JM, Aung N, Rayner JJ, Werys K, Fung K, Puchta H, Sanghvi MM, Moon NO, Thomson RJ, Thomas KE, Robson MD, Grau V, Petersen SE, Neubauer S and Piechnik SK. Automated localization and quality control of the aorta in cine CMR can significantly accelerate processing of the UK Biobank population data. PLoS One. 2019;14:e0212272.

5. Kitt J, Frost A, Mollison J, Tucker KL, Suriano K, Kenworthy Y, McCourt A, Woodward W, Tan C, Lapidaire W, Mills R, Khan M, Tunnicliffe EM, Raman B, Santos M, Roman C, Hanssen H, Mackillop L, Cairns A, Thilaganathan B, Chappell L, Aye C, Lewandowski AJ, McManus RJ and Leeson P. Postpartum blood pressure self-management following hypertensive pregnancy: protocol of the Physician Optimised Post-partum Hypertension Treatment (POP-HT) trial. *BMJ Open*. 2022;12:e051180.

6. World Health Organization (WHO): Introduction to Drug Utilization Research. Chapter 6: Drug Utilization Metrics and Their Applications. 2003.

**Table S1: Characteristics of participants included in CMR aortic imaging analysis**

| **Parameter, *Unit*** | **Intervention (n = 80)** | **Usual Care (n = 65)** |
| --- | --- | --- |
| **Patient characteristics** |  |  |
| Mean age, y (SD) | 34.2 (5.1) | 33.0 (5.2) |
| Mean booking BMI, kg/m² (SD) | 28.1 (5.5) | 29.2 (8.0) |
| Mean booking height, cm (SD) | 165.8 (6.7) | 164.1 (6.5) |
| Mean booking BSA, m² (SD) | 1.9 (0.2) | 1.9 (0.3) |
| Mean systolic blood pressure at first antenatal visit, mmHg (SD) | 118.7 (10.1) | 116.9 (10.3) |
| Mean diastolic blood pressure at first antenatal visit, mmHg (SD) | 73.7 (9.4) | 71.9 (8.6) |
| Pre-pregnancy smoking reported^*^, No. (%) | 22 (23.7) | 24 (29.6) |
| IMD quintile^†^, median (IQR) | 2 (1, 3) | 1 (1, 2) |
| **Race and ethnicity^**^, n (%)** |  |  |
| Asian | 7 (7.5) | 6 (7.4) |
| Hispanic or Latino | 4 (4.3) | 4 (4.9) |
| Non-Hispanic Black | 6 (6.5) | 3 (3.7) |
| Non- Hispanic White | 76 (81.7) | 67 (82.7) |
| Pacific Islander | 0 (0.0) | 1 (1.2) |
| **Pregnancy characteristics** |  |  |
| Pre-eclampsia^‡^, No. (%) | 59 (63.4) | 52 (64.2) |
| Gestational hypertension^‡^, No. (%) | 34 (36.6) | 29 (35.8) |
| HELLP syndrome subset of pre-eclampsia^‡^, No. (%) | 4 (4.3) | 1 (1.2) |
| Median duration of ante-natal antihypertensive treatment, days (IQR) | 4.0 (2.0, 16.0) | 5.0 (1.0, 17.5) |
| Early diagnosis of pre-eclampsia or gestational hypertension ≤ 33 weeks and 6 days gestation, No.  (%) | 19 (20.4) | 19 (23.5) |
| Median gestation at delivery, wks (IQR) | 39.5 (37.2, 40.3) | 39.0 (37.0, 40.3) |
| Primiparous, No. (%) | 57 (61.3) | 58 (71.6) |
| Previous hypertensive pregnancy, No. (%) | 27 (29.0) | 8 (9.9) |
| Assisted reproduction pregnancy, No. (%) | 6 (6.5) | 9 (11.1) |
| Multi-fetal pregnancy, No. (%) | 4 (4.3) | 4 (4.9) |
| Spontaneous vaginal birth (%) | 38 (40.9) | 23 (28.4) |
| Assisted vaginal birth (%) | 15 (16.1) | 19 (23.5) |
| Emergency Caesarean section^§^ (%) | 36 (38.7) | 35 (43.2) |
| Elective Caesarean section^§^ (%) | 4 (4.3) | 4 (4.9) |
| Fetal growth restriction^**,\|\|^, No. (%) | 23 (24.7) | 26 (32.1) |
| Neonatal unit admission^g^, No. (%) | 20 (21.5) | 24 (29.6) |
| Mean birthweight, kg (SD) | 3.1 (0.8) | 3.0 (0.9) |

^*^Smoking prior to pregnancy for a > 12-month period ^†^IMD refers to the Index of Multiple Deprivation, a measure of socioeconomic disadvantage defined in quintiles with 1 describing the least deprived and 5 the most deprived. Data from n=213 (intervention n=109, usual care n=104); ^‡^Classification as gestational hypertension, pre-eclampsia and HELLP syndrome were based on definitions provided in the NICE guideline (NG 133) “Hypertension in pregnancy; diagnosis and management”, definitions for which can be found in the protocol provided in the supplementary material.

^§^Category of caesarean section was defined as per NICE guidance on Caesarean birth (NG 192). The term “Elective Caesarean” refers to an electively scheduled caesarean timed to suit the patient or health care provider. “Emergency Caesarean” spans the categories of “no maternal or fetal compromise but needs early birth” to “immediate threat to the life of the patient or fetus”. ^||^ IUGR defined as a fetus whose weight was <10^th^ percentile for its gestational age postpartum; ^¶^A Neonatal Unit is a part of a hospital which provides care for babies who are born prematurely (before 37 weeks' gestation) and is used as an umbrella term here to includes the neonatal intensive care unit, high dependency unit and special care baby unit.

^**^In accordance with UK recommendations, self-reported ethnicity was recorded using standard descriptions derived from those used by UK Office for National Statistics

**Table S2: Diet and Lifestyle characteristics at 9 months postpartum of those participants in the POP-HT trial who had both Vicorder® and Aortic magnetic resonance imaging.**

| **Parameter, unit** | **Overall  *(n = 145)*** | | **Intervention  *(n = 80)*** | | **Control  *(n = 65)*** | |
| --- | --- | --- | --- | --- | --- | --- |
| **Postnatal characteristics** | ***n*** |  | ***n*** |  | ***n*** |  |
| Mean BMI at 9 months, kg/m² (SD) | 145 | 29.6 (7.1) | 80 | 28.9 (5.8) | 65 | 30.5 (8.4) |
| Breastfeeding at 9 months, n (%) | 145 | 75 (51.7) | 80 | 44 (55.9) | 65 | 31 (47.7) |
| **Activity, hours/day** | **n** | **Median** ± **IQR** | **n** | **Median** ± **IQR** | **n** | **Median** ± **IQR** |
| Sleep | 127 | 9.12 ± 1.3 | 70 | 9.12 ± 1.7 | 57 | 9.12 ± 1.3 |
| Sedentary | 127 | 8.88 ± 1.9 | 70 | 9 ± 2.4 | 57 | 8.64 ± 2.2 |
| Light activity | 127 | 0.48 ± 0.5 | 70 | 0.48 ± 0.7 | 57 | 0.48 ± 0.7 |
| Walking | 127 | 0.72 ± 0.7 | 70 | 0.72 ± 0.7 | 57 | 0.72 ± 0.7 |
| Moderate activity | 127 | 4.80 ± 2.4 | 70 | 4.80 ± 2.4 | 57 | 4.80 ± 2.2 |
| **Smoking history** | ***n*** | ***n (%)*** | ***n*** | ***n (%)*** | ***n*** | ***n (%)*** |
| Non-smoker | 140 | 97 (69.3) |  | 117 (70.5) |  | 130 (84.0) |
| Ex-smoker | 140 | 39 (27.8) | 78 | 21 (26.9)* | 62 | 8 (12.9)* |
| Current Smoker | 140 | 4 (2.9) | 78 | 2 (2.6) | 62 | 2 (3.2) |
| **Alcohol consumption, units/week** | ***n*** | ***n (%)*** | ***n*** | ***n (%)*** | ***n*** | ***n (%)*** |
| 0 | 139 | 89 (64.0) | 78 | 50 (64.1) | 61 | 39 (63.9) |
| 1-7 | 139 | 42 (30.2) | 78 | 22 (28.2) | 61 | 20 (32.8) |
| 8-14 | 139 | 5 (3.6) | 78 | 5 (6.4) | 61 | 0 (0) |
| 15+ | 139 | 3 (2.2) | 78 | 1 (1.3) | 61 | 2 (3.3) |
| **Salt intake** | ***n*** | ***n (%)*** | ***n*** | ***n (%)*** | ***n*** | ***n (%)*** |
| Low | 138 | 33 (23.9) | 77 | 20 (26.0) | 61 | 13 (21.3) |
| Moderate | 138 | 78 (56.5) | 77 | 42 (54.5) | 61 | 36 (59.0) |
| High | 138 | 27 (19.6) | 77 | 15 (19.5) | 61 | 12 (19.7) |
| **Contraception** | ***n*** | ***n (%)*** | ***n*** | ***n (%)*** | ***n*** | ***n (%)*** |
| None | 145 | 89 (61.4) | 80 | 48 (60.0) | 65 | 41 (63.0) |
| Progesterone only pill | 145 | 30 (20.7) | 80 | 19 (23.8) | 65 | 11 (17.0) |
| Combined oral contraceptive pill | 145 | 7 (4.8) | 80 | 3 (3.8) | 65 | 4 (6.1) |
| Implant | 145 | 4 (2.8) | 80 | 2 (2.5) | 65 | 2 (3.2) |
| Coil | 145 | 12 (8.3) | 80 | 5 (6.3) | 65 | 7 (10.7) |
| Depot injection | 145 | 3 (2.0) | 80 | 3 (3.6) | 65 | 0 (0) |

^*^Differences were statistically tested using *X^2^* analysis and no statistically significant differences between randomization arms found except for smoking history where was a greater proportion of ex-smokers in the intervention arm (chi-square statistic is 6.1727. The *p*-value is .045668. The result is significant at *p* < .05). Alcohol intake did not differ when categorized into low (0-7), moderate (8-14) and high(>15).

**Table S3: Details of anti-hypertensive use throughout the POP-HT trial**


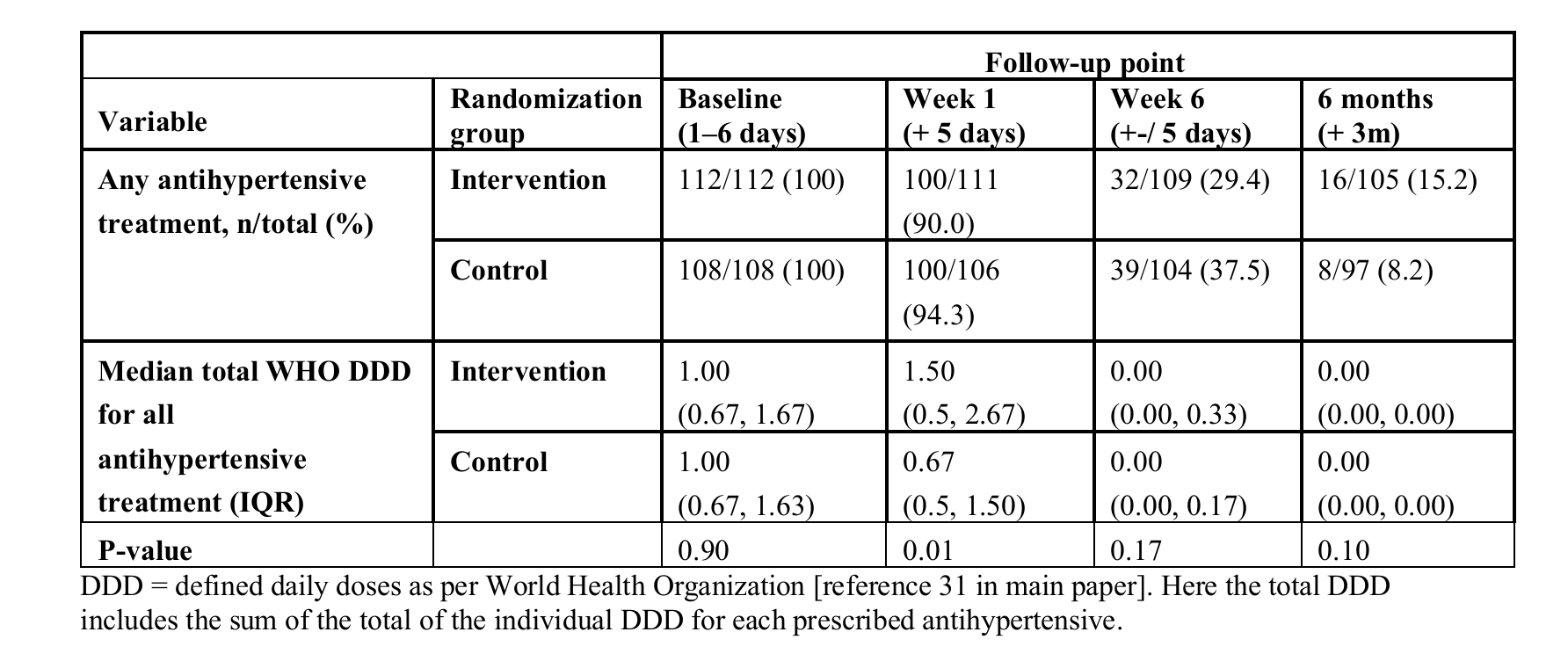


DDD refers to the defined daily doses as per the World Health Organization (WHO).^6^It is described as the assumed average maintenance dose per day for a drug used for its main indication in adults. Here the total DDD includes the sum of the total of the individual DDD for each prescribed antihypertensive

**Table S4: Unadjusted Vicorder, clinic blood pressure, and ambulatory blood pressure data from baseline to final study visit in POP-HT Vascular Imaging Sub-Study cohort**

|  | **Intervention** | | **Usual care** | | **Unadjusted regression coefficients** | | |
| --- | --- | --- | --- | --- | --- | --- | --- |
|  | **Baseline**^†^  **Mean (SD)** | **V4**^‡^  **Mean (SD)** | **Baseline**^†^  **Mean (SD)** | **V4**^‡^  **Mean (SD)** | **Difference** | **95% C.I.*** | **P value** |
| **Vicorder® Parameter** | | | | | | | |
| Aortic SBP, mmHg | 134.2 (13.5) | 123.9 (12.7) | 133.7 (15.4) | 125.9 (13.6) | -1.9 | -5.6 to 1.9 | 0.325 |
| Aortic DBP, mmHg | 75.9 (10.1) | 71.0 (9.7) | 74.5 (10.1) | 76.1 (9.9) | -5.2 | -8.0 to -2.4* | <0.001 |
| Augmentation index (%) | 27.6 (10) | 6.72 (6.5) | 26.1 (11) | 7.48 (7.3) | -0.86 | -2.82 to 1.10 | 0.390 |
| PWV (m/s) | 11.8 (2.5) | 12.7 (2.5) | 11.4 (2.0) | 13.3 (2.7) | -0.7 | -1.4 to 0.0 | 0.067 |
| **Clinic Blood Pressure** | | | | | | | |
| Mean SBP, mmHg | 129.1 (13.2) | 126.2 (10.5) | 127.1 (12.6) | 129.3 (11.5) | -3.09 | -6.75 to 0.57 | 0.097 |
| Mean DBP, mmHg | 83.6 (10.5) | 82.4 (7.8) | 80.5 (10.5) | 84.6 (8.6) | -2.13 | -4.87 to 0.61 | 0.127 |
| **24-Hour Ambulatory Blood Pressure** | | | | | | | |
| Mean 24-hour average SBP, mmHg |  | 114.0 (7.9) |  | 120.0 (9.3) | -5.68 | -8.58 to -2.78* | <0.001 |
| Mean 24-hour average DBP, mmHg |  | 71.5 (5.6) |  | 76.2 (5.9) | -4.64 | -6.56 to -2.72* | <0.001 |
| Mean diurnal average SBP, mmHg |  | 118.2 (7.7) |  | 123.9 (9.2) | -5.67 | -8.54 to -2.81* | <0.001 |
| Mean diurnal average DBP, mmHg |  | 75.8 (5.6) |  | 80.1 (6.3) | -4.24 | -6.23 to -2.25* | <0.001 |

Parametric: mean (SD). SBP, systolic blood pressure; DBP, diastolic blood pressure; PWV, pulse wave velocity. * Signifies 95% confidence intervals of mean difference which do no cross zero. ^†^Baseline visit took place between days 1-6 postpartum on the postnatal ward. ^‡^V4 (4^th^) and final study visit which took place between 6-12 months postpartum (mean 258 days; 258.9 intervention and 257.4 usual care). Baseline 219 participants; intervention n=111, usual care n=108; V4 194 participants; intervention n= 102, usual care n=92. Unadjusted mean differences, 95% confidence intervals, and p-values are given for all parameters, using parametric tests to assess differences between usual care and intervention groups.

**Table S5: Sensitivity analysis removing those participants who remained on anti-hypertensives at the time of V4 to assess its impact on aortic distensibility**

|  |  |  | Unadjusted model | | Adjusted model 1 | | Adjusted model 2 | |
| --- | --- | --- | --- | --- | --- | --- | --- | --- |
| Group | n | Mean (SD) | Coeff. (95%CI) | P value | Coeff. (95%CI) | P value | Coeff. (95%CI) | P value |
| Usual Care | 57 | 5.81 (2.31) | 0.92 (0.16 to 1.69) | 0.018 | 1.066 (0.32 to 1.82) | 0.006 | 0.99 (0.22 to 1.75) | 0.012 |
| Intervention | 67 | 6.74 (1.98) |  |  |  |  |  |  |

Model 1 adjusted for age and BSA; Model 2 adjusted for age, BSA and V4_Mean_Diastolic BP

**Table S6: Aortic distensibility assessed by cardiac magnetic resonance imaging of the ascending aorta at ~9 months postpartum using pulse pressure derived from Vicorder at V4**

|  |  |  | Unadjusted model | | Adjusted model 1 | | Adjusted model 2 | |
| --- | --- | --- | --- | --- | --- | --- | --- | --- |
| Group | n | Mean (SD) | Coeff. (95%CI) | P value | Coeff. (95%CI) | P value | Coeff. (95%CI) | P value |
| Control | 65 | 5.56 (2.33) | 1.26 (0.57 to 1.95) | <0.001 | 1.39 (0.71 to 2.07) | <0.001 | 1.33 (0.65 to 2.01) | <0.001 |
| Intervention | 80 | 6.82 (1.88) |  |  |  |  |  |  |

Model 1 adjusted for age and BSA; Model 2 adjusted for age, BSA and V4_Mean_Diastolic BP

**Table S7: Summary of POP-HT trial outcomes as described in the published protocol paper. (The pre-defined outcomes for the vascular sub-study are highlighted in red)**

|  | Objectives | Outcome Measures | Timepoint(s) |
| --- | --- | --- | --- |
| Primary | To compare postpartum diastolic BP in the intervention arm to the control arm. | 24-hour average diastolic BP measured by assessed by SPACELAB 90217 24hr Ambulatory blood pressure monitoring (ABPM) | 6-9 months postpartum |
| Secondary | To compare the effect of the intervention on cardiovascular, cerebrovascular and vascular phenotypes | **BP based**   1. 24 hr. average systolic blood pressure assessed by SPACELAB 90217 24hr ABPM 2. Mean diurnal diastolic blood pressure assessed by SPACELAB 90217 ABPM 3. Mean diurnal systolic blood pressure assessed by SPACELAB 90217 ABPM 4. Mean nocturnal diastolic blood pressure assessed by SPACELAB 90217 24hr ABPM 5. Mean nocturnal systolic blood pressure assessed by SPACELAB 90217 24hr ABPM 6. Mean clinic diastolic blood pressure measured during study visit (mean of 2+3) 7. Mean clinic systolic blood pressure measured during study visit (mean of 2+3)   **Cardiac MRI**   1. Left ventricular (LV) mass indexed to end-diastolic volume and body surface area (BSA) 2. LV EDV indexed to BSA 3. LV wall thickness 4. LA volume indexed to BSA 5. Right ventricular (RV) mass indexed to end-diastolic volume and body surface area (MRI) 6. RV EDV indexed to BSA 7. RA volume indexed to BSA 8. LV ejection fraction (EF) & RV EF 9. LV and RV stroke volumes indexed to BSA 10. Myocardial fibrosis 11. ECV (extra-cellular volume)   **Echo**   1. LV Diastolic function:   E/E’ average, E/A ratio, E deceleration time   1. Global longitudinal strain (GLS) 2. LV systolic function (EF by Biplane Simpson’s) 3. LA volume by Biplanar assessment   **Vascular**:   1. Pulse wave velocity 2. Augmentation index 3. Aortic BP 4. Aortic distensibility (MRI) | Week 6 and 6-9 months for the 24 hr ABPM data  Baseline, week 1, week 6 and 6-9 months for the clinic blood pressures  For Cardiac MRI at 6-12 months postpartum  At baseline and at 6-12 months postpartum for Echo outcome measures  PWV, Aortic BP & AI at baseline and at 6-12 months  Aortic distensibility at 6-12 months (MRI) |

**Figure S1 below demonstrates an image of pulse wave analysis using a Vicorder®. Brachial artery pressure waveforms are used to derive central blood pressure.**

**Figure S2 below demonstrates a trace obtained from both brachial (top trace) and femoral cuff (bottom trace) using a Vicorder®, from which the pulse wave velocity (PWV) is then derived when the distance between the two cuffs is provided to the software.**

**
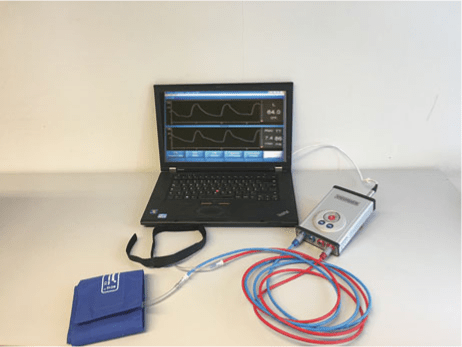
**

**Figure S3 below shows an example of the automated image analysis software (Matlab, Mathworks, Inc, MA) developed by Biasiolli et al and validated on the UK BioBank dataset^4^ for assess images suitable for distensibility analysis such as that described in this paper**


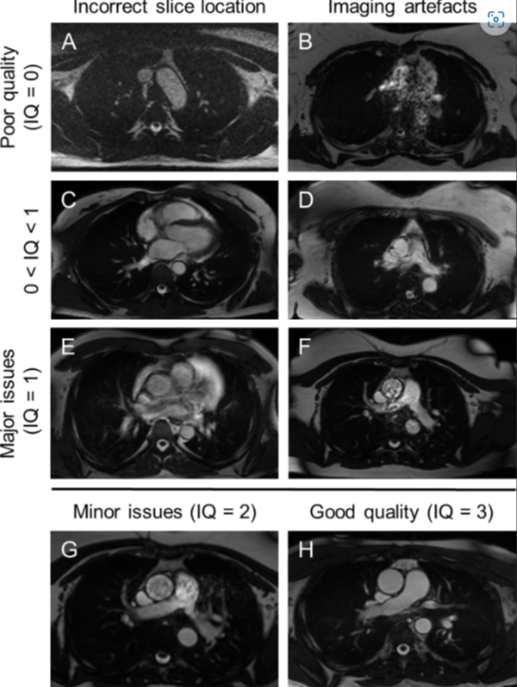

Supplement: Supplementary file 1 [file hyp-82-1938-s001.docx]
